# Deletion of cyclooxygenase-2 inhibits K-ras–induced lung carcinogenesis

**DOI:** 10.18632/oncotarget.5558

**Published:** 2015-10-03

**Authors:** Yong Pan, Yan Jiang, Lin Tan, Murali K. Ravoori, Mihai Gagea, Vikus Vikas Kundra, Susan M. Fischer, Peiying Yang

**Affiliations:** ^1^ Department of Palliative, Rehabilitation and Integrative Medicine, The University of Texas MD Anderson Cancer Center, Houston, TX, USA; ^2^ Department of Cancer Systems Imaging, The University of Texas MD Anderson Cancer Center, Houston, TX, USA; ^3^ Department of Veterinary Medicine & Surgery, The University of Texas MD Anderson Cancer Center, Houston, TX, USA; ^4^ Department of Diagnostic Radiology, The University of Texas MD Anderson Cancer Center, Houston, TX, USA; ^5^ Department of Molecular Carcinogenesis, The University of Texas MD Anderson Cancer Center, Houston, TX, USA

**Keywords:** COX-2, K-ras oncogene, lung adenocarcinoma, mouse, MAP kinase

## Abstract

The purpose of this study was to identify the role COX-2 plays in K-ras–induced lung carcinogenesis. We crossed COX-2–homozygous knockout mice with K-ras^LA1^ (G12D) expressing mice to obtain COX-2–deficient mice with K-ras expression (K-ras/COX-2^−/−^ mice) and COX-2 wild type mice with K-ras expression (K-ras mice). At 3.5 months of age, the K-ras/COX-2^−/−^ mice had significantly fewer lung adenocarcinomas and substantially smaller tumors than K-ras mice. K-ras/COX-2^−/−^ mice also had significantly fewer bronchioalveolar hyperplasias than K-ras mice. Compared with lung tumors from K-Ras mice, the levels of prostaglandin E_2_ (PGE_2_) were significantly lower, whereas levels of the PGE_2_ metabolite 13,14-dihydro-15-keto-PGE_2_ were significantly higher, in lung tumors from K-ras/COX-2^−/−^ mice. In addition, K-ras/COX-2^−/−^ mice had strikingly lower rates of tumor cell proliferation and expressed less MEK and p-Erk1/2 protein than K-ras mice did. In line with this, knocking down COX-2 in mutant K-ras non-small cell lung cancer A549 cells reduced colony formation, PGE_2_ synthesis and ERK phosphorylation compared to that of vector control cells. Taken together, these findings suggest that COX-2 deletion contributes to the repression of K-ras–induced lung tumorigenesis by reducing tumor cell proliferation, decreasing the production of PGE_2_, and increasing the production of 13,14-dihydro-15-keto-PGE_2_, possibly via the MAPK pathway. Thus, COX-2 is likely important in lung tumorigenesis, and COX-2 and its product, PGE_2_, are potential targets for lung cancer prevention.

## INTRODUCTION

The proto-oncogene *KRAS*, coding a membrane-associated GTPase signaling protein that regulates proliferation, differentiation, and cell survival, is mutated in 30–50% of lung adenocarcinomas, the most common histological subtype of non–small cell lung cancer (NSCLC) [1–3]. Studies suggest that *KRAS* mutations play significant roles in the initiation and progression of lung cancer and contribute to the poor prognosis of the disease. Researchers have focused on inhibiting the *KRAS* gene by either using a genetic engineer approach [4] or blocking the posttranslational modifications required for K-ras activation. However, despite numerous studies, the mechanism by which mutated *KRAS* exerts its effects in lung cancer is unclear.

Cyclooxygenase (COX) is the key enzyme in the conversion of arachidonic acid to prostaglandins (PGs), which are known to promote tumor growth, angiogenesis, and metastasis [3]. COX-2 is overexpressed at most stages of lung tumor progression, including in hyperplastic bronchial epithelium, atypical adenomatous hyperplasia and metastatic lung cancer [5–7]. Hida et al. found that COX-2 is expressed in one-third of atypical adenomatous hyperplasias and carcinomas *in situ*, which again suggests that COX-2 plays an important role throughout the progression of lung cancer, from pre-malignant lesion to metastatic phenotype [5]. Recent studies have demonstrated that the transfection of constitutively active *KRAS* mutant into E10 cells upregulates COX-2 [8]. Consistent with its expression patterns in human lung cancer, COX-2 is also expressed in rodent lung tumor, and transgenic COX-2 overexpression can drive tumorigenesis in mouse lung [9]. In mice, *KRAS* mutations are found in >90% of spontaneous and chemically-induced lung tumors [10]. In the pulmonary microenvironment, many stimuli associated with lung cancer risk, such as transforming growth factor-β1 and epidermal growth factor, can induce COX-2 expression [11], which has been found to correlate with poor prognosis. However, the role of COX-2 in lung tumorigenesis in mice carrying the *KRAS* oncogene remains unknown.

In the present study, we used a knockout strategy to investigate the role of COX-2 in mediating *KRAS* mutation–induced lung tumorigenesis and the effect of COX-2 deficiency on lung morphology and tumorigenesis in mice. Our results suggest that the COX-2 pathway plays an important role in *KRAS-*driven lung tumorigenesis, probably mediated through the mitogen-activated protein kinase (MAPK) pathway.

## RESULTS

### COX-2 deletion inhibits K-ras-induced lung tumor growth

To assess whether knockout of COX-2 affected lung tumorigenesis in mice carrying a *K-ras* allele (G12D) that spontaneously developed multifocal lung adenocarcinomas, we generated the K-ras/COX-2^−/−^ mice by breeding female K-ras/COX-2^+/−^ with male COX-2–null mice. Our first goal was to determine the time of tumor development in these mice to identify a suitable time at which to subject the mice to the experiment. We found that lung nodules less than 1 mm diameter (adenomas) were first detectable at 4–5 weeks, whereas larger lung nodules (>1mm) (adenocarcinomas) appeared after 13 weeks. Compared with K-ras mice, K-ras/COX-2^−/−^ mice had fewer lung tumor nodules at both 2 and 4 months of age.

**Figure 1:**
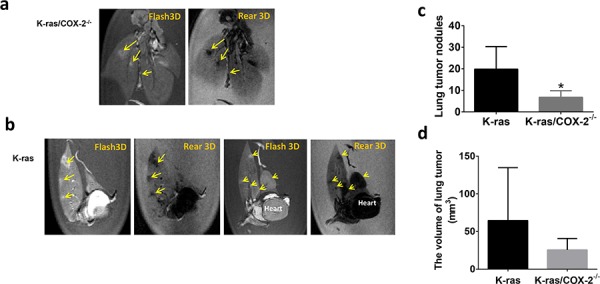
Knocking out COX-2 in mice with K-ras mutation significantly reduced the lung tumor development **a.** *Ex vivo* MRI revealed fewer lung nodules in (a) 4-month-old K-ras/COX-2^−/−^ mice than in **b.** 4-month-old K-Ras mice. Yellow arrows indicated lung tumor nodules in mice; **c.** The mean number of lung nodules in 3.5-month-old K-ras mice was significantly higher than that in K-ras/COX-2^−/−^ mice, as assessed using *ex vivo* MRI; **d.** The mean lung tumor volume of 3.5-month-old K-ras mice was higher than that of 3.5-month-old K-ras/COX-2^−/−^ mice. Data in panels (c) and (d) are means ± SDs; *p < 0.05.

To accurately determine the number and size of tumors in each mouse strain, we performed *ex vivo* 3-dimensional (3D) magnetic resonance imaging (MRI) on perfused lung tissues in a blinded fashion. *Ex vivo* 3D MRI revealed that compared with 4-month-old K-ras mice, 4-month-old K-ras/COX-2^−/−^ mice had fewer lung tumor nodules (6 vs 22) and smaller tumors (Figures 1a and 1b). In line with this, the mean lung tumor incidence in 3.5-month-old K-ras/COX-2^−/−^ mice (6.75 tumors/mouse) was significantly lower than that of K-ras mice (19.83 tumors/mouse; *p* < 0.05) (Figure 1c). Similarly, the mean tumor size of K-ras mice (65.58 ± 31.11 mm^3^) was much higher than that of K-ras/COX-2^−/−^ mice (25.53 ± 8.66 mm^3^) (Figure 1d). The results show that the deletion of the COX-2 gene inhibits the development of lung adenocarcinoma in mice carrying mutant K-ras.

### COX-2 deletion reduces lung hyperplasia and intraepithelial neoplasia development

**Figure 2:**
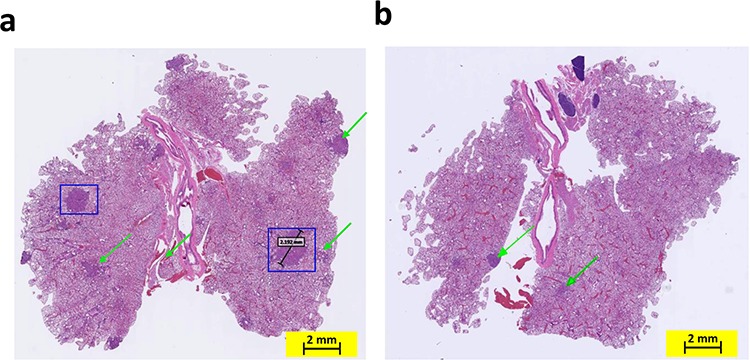
Knocking out the COX-2 gene in K-ras mice inhibited the formation of adenocarcinoma in the lungs by age 4 months Staining with hematoxylin and eosin revealed more adenomas (indicated with green arrows) and adenocarcinomas (outlined with blue squares) in K-ras mouse lungs **a.** than in K-ras/COX-2^−/−^ mouse lungs **b.** at age 4 months.

**Figure 3:**
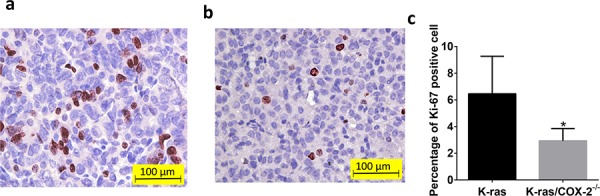
COX-2 deletion reduced the proliferative potential of lung tumor cells Ki-67 staining revealed more proliferating cells in **a.** lung tumor from K-ras/COX-2^−/−^ mice than in **b.** lung tumor from K-ras mice. **c.** The mean percentage of Ki-67 positive cells in lung tumor from K-ras mice was significantly higher than that in lung tumor from K-ras/COX-2^−/−^ mice. Data are means ± SDs.

COX-2 overexpression has been reported in both hyperplastic bronchial epithelium and atypical adenomatous hyperplasia [12]. Therefore, we sought to determine whether COX-2 deletion affects the incidence of bronchial hyperplasia, bronchial adenoma, and/or bronchial carcinoma in mice carrying mutant *KRAS*. Histopathological analysis demonstrated that the lung tissues of K-ras mice had significantly more bronchioalveolar hyperplasia than did lung tissues of K-ras/COX-2^−/−^ mice. The mean incidence of bronchioalveolar hyperplasia of K-ras mice (9.5 ± 3.2 lesions/mouse) was significantly higher than that of K-ras/COX-2^−/−^ mice (5.3 ± 2.5 lesions/mouse; *p* < 0.05). Similarly, the mean incidence of bronchioalveolar adenoma of K-ras mice (5.5 ± 4.4 tumors/mouse) was higher than that of K-ras/COX-2^−/−^ mice (2.3 ± 1.0 tumors/mouse). This trend was further illustrated by the hematoxylin and eosin staining of lung tissues from K-ras and K-ras/COX-2^−/−^ mice (Figure 2). We also found alveolar inflammation between the tumors and alveolar layer in some mice, but the difference between K-ras and K-ras/COX-2^−/−^ mice was not significant in this regard. The incidence of bronchioalveolar carcinoma in K-ras/COX-2^−/−^ mice was also markedly lower than that of K-ras mice, but the difference between the two groups of mice was not significant (Table 1). Ki67 staining indicated that the proliferative potential of lung tumor cells in K-ras/COX-2^−/−^ mice (Figure 3a) was lower than that in K-ras mice (Figure 3b) evidenced by the percentage of tumor cells with Ki-67–positive staining in K-ras/COX-2^−/−^ mice (2.9%) was significantly lower than that inK-ras mice (6.2%; *p* < 0.05) (Figure 3c).

**Table 1:** Incidence of lung lesions by histopathological subtype among K-ras and K-ras/COX-2^−/−^ mice

Mouse Strain	Bronchioalveolar Carcinoma	Bronchioalveolar Adenoma	Bronchioalveolar Hyperplasia
K-ras/COX-2^−/−^	0.2 ± 0.4	2.3 ± 1.0	5.3 ± 2.5*
K-ras	0.8 ± 1.0	5.5 ± 4.4	9.5 ± 3.2

Note: Data are means ± standard deviations.

* *p* < 0.05

### COX-2 deletion inhibits PGE_2_ production and metabolism

**Figure 4:**
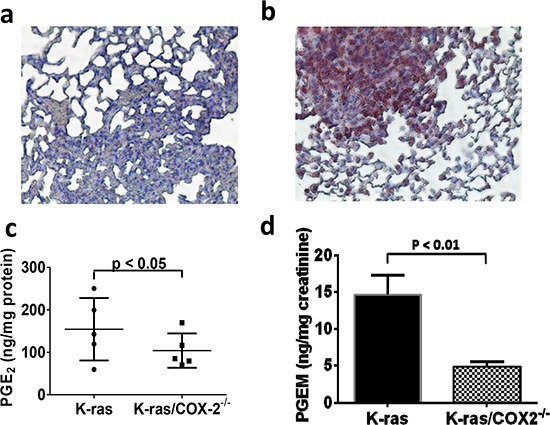
COX-2 knockdown was confirmed by the much lower positive COX-2 signaling in **a.** K-ras/COX-2^−/−^ mice compared with **b.** K-ras mice. In line with this finding, concentrations of the COX-2 metabolite PGE_2_ in **c.** lung tumor nodules and PGE_2_ metabolite in **d.** urine from K-ras/COX-2^−/−^ mice were significantly lower than those in K-ras mice. Data are means ± SDs.

COX-2 is a rate-limiting enzyme in prostaglandin biosynthesis. Thus, one predicted consequence of COX-2 downregulation is a decrease in prostaglandin synthesis. To assess the potential changes in lung tissue metabolism brought about by COX-2 downregulation *in vivo*, we first used immunohistochemical staining to verify expression of the COX-2 protein in the lung tissue from K-ras and K-ras/COX-2^−/−^ mice. We then assessed the prostaglandin concentrations in lung tissue from K-ras/COX-2^−/−^ and K-ras mice. Lung tissues from K-ras mice had strong COX-2 protein expression (Figure 4a), whereas lung tissues from K-ras/COX-2^−/−^ mice had limited COX-2 protein expression (Figure 4b). Similarly, PGE_2_ levels in lung tumor tissue from K-ras/COX-2^−/−^ mice were significantly lower than those in lung tumor tissue from K-ras mice (Figure 4c). Similarly, levels of tetranor-PGEM, a urinary metabolite of PGE_2_, in K-ras mice were 3 times higher than in K-ras/COX-2^−/−^ mice (*p* < 0.05) (Figure 4d). The level of other cyclooxygenase products, such as 6-keto-PGF_1α_ or thromboxane B_2_ (TXB_2_) were also reduced in the lung tumor tissues of K-ras/COX-2^−/−^ mice compared to that of K-ras mice (Figure S1.)

**Figure 5:**
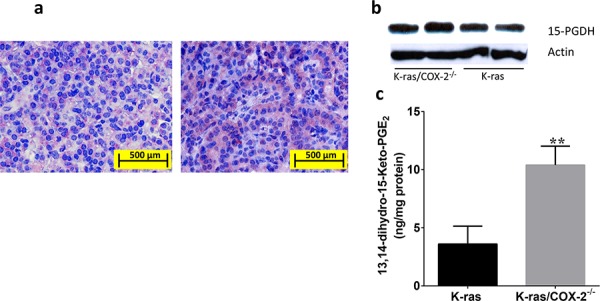
Knockdown of COX-2 increased the expression of 15-PGDH and formation of the PGE_2_ metabolite 13,14-dihydro-15-keto-PGE_2_ in lung tissue **a.** Immunohistochemical staining revealed 15-PGDH in the lung tissue of K-ras mice (left panels) and K-ras/COX-2^−/−^ mice (right panels). **b.** Western blot analysis confirmed protein expression of 15-PGDH in the lung tissues of K-ras/COX-2^−/−^ mice and K-ras mice. **c.** The mean concentration of 13,14-dihydro-15-keto-PGE_2_ in the lung tumor nodules of K-ras/COX-2^−/−^ mice was higher than that in the lung tumor nodules of K-ras mice. Data are means ± SDs; **p < 0.01.

In gastric cancer cells, COX-2 deletion upregulates 15-hydroxyprostaglandin dehydrogenase (15-PGDH), an enzyme responsible for PGE_2_ degradation [13]. Therefore, we determined the expression of this enzyme and its arachidonate metabolite 13,14-dihydro-15-keto-PGE_2_ in lung tumor tissues from K-ras and K-ras/COX-2^−/−^ mice. Immunohistochemical staining (Figure 5a) and Western blotting (Figure 5b) revealed that the protein expression of 15-PGDH in the lung tissues of K-ras/COX-2^−/−^ mice was higher than that in the lung tissues of K-ras mice. In line with this finding, the levels of 13,14-dihydro-15-keto-PGE_2_ in lung tumor tissue from K-ras/COX-2^−/−^ mice were almost 3 times higher than in lung tumor tissue from K-ras mice (Figure 5c, *p* < 0.01). This suggests that the reduction of PGE_2_ in lung tumor tissues is due to both the decrease in PGE_2_ synthesis and the increase of PGE_2_ degradation.

### COX-2 knockdown altered the MAPK pathway in lung tumor tissues

**Figure 6:**
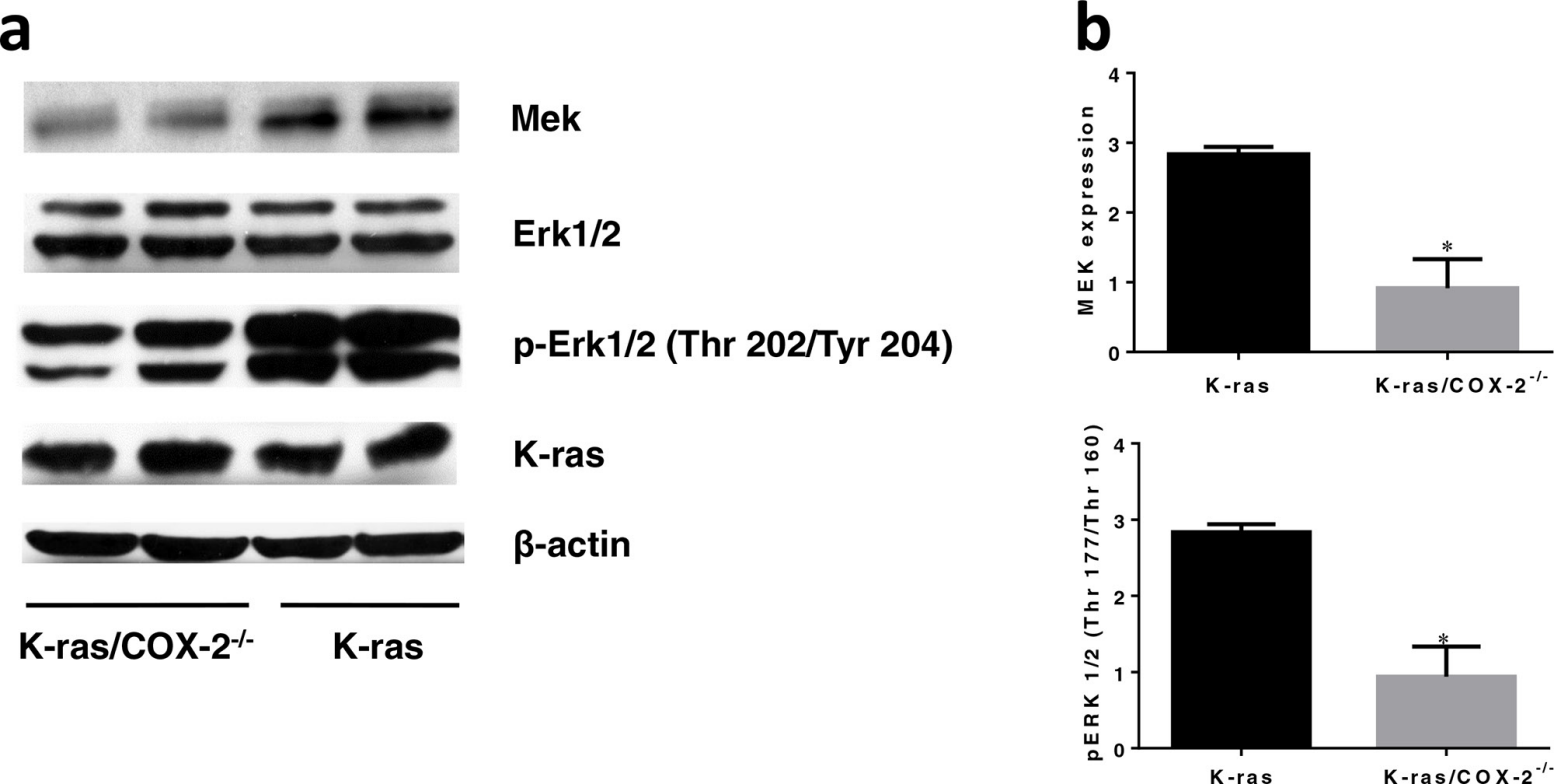
COX-2 deletion led to downregulation of the MAPK pathway and significantly reduced total MEK and p-Erk1/2 **a.** Western blot images and **b.** quantification revealed protein expression of MEK and p-Erk1/2 were lower in K-ras/COX2^−/−^ mice compared to K-ras mice. Data are means ± SDs; *p < 0.05.

Multiple mechanisms, including the RAS/MAPK/Erk [14, 15] and PI3K/AKT [16] pathways, have been proposed to be associated with PGE_2_-induced cancer cell proliferation. Therefore, we investigated the potential role of COX-2 in the regulation of both the PI3K/AKT and MAPK pathways in lung tissue. We hypothesized that COX-2 is involved in the AKT and MAPK pathways and that this involvement increases cancer cell proliferation and promotes lung cancer. We found that the expression of MEK and P-Erk1/2 proteins in lung tissues from K-ras/COX-2^−/−^ mice were significantly lower than those in lung tissues from K-ras mice (Figure 6). These results suggest that COX-2 deletion may lead to inhibition of the MAPK/Erk pathway, thereby inhibiting cancer cell proliferation and carcinogenesis in lung tissues.

### Knocking down COX-2 in K-ras mutant NSCLC A549 cells reduced the cell proliferation via down-regulation of ERK phosphorylation

**Figure 7:**
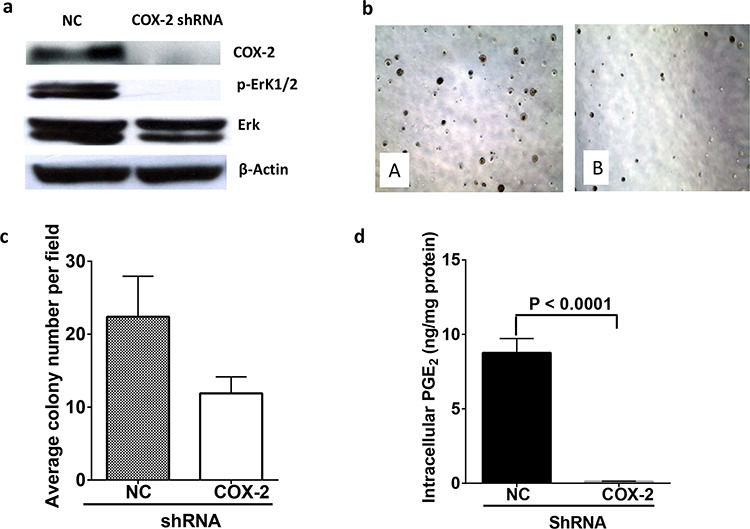
COX-2 knock down reduced colony formation of A549 cells by reduction of PGE_2_ and down regulation of ERK phosphorylation **a.** Protein expression of COX-2, ERK, and pERK in control shRNA or COX-2 shRNA transfected A549 cells. **b.** Microscopic image of colony formation of control shRNA transfected (A) or COX-2 shRNA transfected A549 cells (B). **c.** Quantitative analysis of colony formation in control shRNA or COX-2 shRNA transfected A549 cells. **d.** Intracellular PGE_2_ levels analyzed by LC-MS/MS. Data are mean ± SEM.

Although we and others have shown previously that selective COX-2 inhibitors, such as celecoxib or the omega-3 fatty acid, eicosapentaenoic acid, suppressed proliferation of human NSCLC A549 cells [16], whether knocking down COX-2 gene would affect the proliferation of A549 cells has not been tested. Figure 7 shows that when the COX-2 was down-regulated by transfecting the A549 cells with shRNA of COX-2 (Figure 7a), proliferation of A549 cells was reduced as evidenced by the lower colony formation (Figure 7b and 7c) than that of control shRNA transfected A549 cells. The production of PGE_2_ was significantly reduced in shRNA COX-2 transfected A549 cells compared to vector transfected A549 cells (Figure 7d). Additionally, the levels of other cyclooxygenase products, such as PGF_2α_ and PGD_2_ were also markedly decreased in the COX-2 knockdown A549 cells (Figure S2). Intriguingly, the phosphorylation of ERK was markedly suppressed by knocking down COX-2 whereas minimum changes in total ERK was observed in A549 cells.

## DISCUSSION

To our knowledge, our study is the first to suggest that the induction of NSCLC by K-ras overexpression is at least partially mediated through the COX-2 pathway. Using a gene knockout strategy, we demonstrated that genetic ablation of the COX-2 gene protects against K-ras-induced lung neoplasia, by decreasing hyperplastic bronchial epithelium and atypical adenomatous hyperplasia in K-ras/COX-2^−/−^ mice. Consistent with previous reports [17], our proliferation assay demonstrated that the percentage of Ki67-positive signals in K-ras/COX-2^−/−^ mice was significantly lower than that in K-ras mice, which suggests that COX-2 is essential to lung tumor development. Our results suggest that COX-2 overexpression is an early event in K-ras-induced lung carcinogenesis.

The present study's findings elucidate the way in which COX-2 metabolism fosters the development of K-ras–induced lung cancer. Smakman et al. demonstrated that mutant KRAS^G12D^ is the primary cause of high levels of COX-2 enzymes and PGE_2_ production in C26 colorectal cancer liver metastases [18]. More recently, Wang et al. reported that transfecting lung epithelial cells with oncogenic K-ras promotes proliferation and cell junctions by inducing COX-2 and its metabolite, PGE_2_ [19]. The components involved in regulating PGE_2_ production have been recognized to also play a significant role in the regulation of tumor development. For example, PGE_2_ levels in colon or lung tumor tissues that are higher than those in adjacent normal tissues might be attributable to not only the upregulated expression of COX-2 or PGE_2_ synthases but also the reduced expression of the key PGE_2_ degradation enzyme 15-PGDH [20–22]. In fact, in a previous study, we found that the levels of COX-2 and PGE_2_ synthase were commonly higher, and the amount of 15-PGDH frequently lower, in NSCLC compared with adjacent normal lung [22]. A similar phenomenon has been observed in gastric cancer [13]. In the present study, we found that COX-2 deletion not only significantly reduces the production of PGE_2_ but also increases the expression of 15-PGDH and significantly increases the formation of the PGE_2_ metabolite 13,14-dihydro-15-keto-PGE_2_. Whether 13,14-dihydro-15-keto PGE_2_ is critical for the tumor suppression caused by COX-2 deletion deserves further investigation. We also investigated the role of arachidonate-associated bioactive lipids, including COX and lipoxygenase metabolites, in lung adenoma and lung adenocarcinoma in K-ras mice. We found that the PGE_2_ level in adenocarcinoma was 2.3 times higher than that in adenoma of K-ras mice (data not shown). Together, these data suggest that PGE_2_ and its metabolite play essential roles in initiating and regulating lung tumorigenesis.

Furthermore, our findings suggest that COX-2 mediates K-Ras–induced lung cancer through the MAPK pathway. The MAPK/Erk signaling cascade is activated by a wide variety of receptors involved in cell growth and differentiation, including receptor tyrosine kinases, integrins, and ion channels [23, 24]. Extensive data have shown that in certain cancers, PGE_2_ induces cell proliferation, enhances angiogenesis, and promotes invasion and metastasis through the activation of the MAPK and AKT pathways [25–27]. In the present study, we assessed the expression of proteins in the PI3K/AKT pathway, such as pAKT, MAPK, MEK and pErk in lung tumor tissues from both K-ras and K-ras/COX-2^−/−^ mice to identify the molecular mechanisms underlying COX-2′s role in lung tumorigenesis in mice carrying the *KRAS* oncogene. Interestingly, COX-2 deletion in K-ras mice did not appear to inhibit the expression of pAKT (data not shown). In contrast, consistent with previous studies, pErk expression in K-ras/COX-2^−/−^ mice was downregulated compared with that in K-ras mice, which suggests that COX-2 deletion reduced PGE_2_ levels, blocked the MAPK pathway, and subsequently inhibited tumor proliferation in K-ras mice. Given that another study has shown that PGE_2_ stimulates the proliferation of NSCLC cells primarily mediated through activation of MAPK/ERK signaling pathway [28], our findings provide further insight into the mechanism by which COX-2 mediates K-ras-induced lung cancer.

One of the limitations of this study was the small number of mice used in this study due to relatively small penetrance of K-ras/COX-2^−/−^ mice. However, even with 4–6 mice, lung tumorigenesis was dramatically reduced as evidenced by significantly fewer tumor nodules and markedly smaller tumors in COX-2 null K-ras mice compared to that in K-ras mice, suggesting that the COX-2 pathway is important for *KRAS* mutation induced lung tumorigenesis.

In conclusion, our findings suggest that COX-2 is essential to the development of K-ras-induced lung cancer and is a viable therapeutic target. However, selective COX-2 inhibitors are cardiotoxic, which limits their long-term use. Therefore, developing non-cardiotoxic selective COX-2 inhibitors and/or agents that target the enzymes regulating PGE_2_ synthesis and degradation may lead to valuable chemopreventive or -therapeutic approaches for patients with mutant *KRAS* lung cancer.

## MATERIALS AND METHODS

### Mouse breeding strategies

COX-2^−/−^ mice were purchased from Jackson Laboratory (Bar Harbor, ME). K-ras^LA1^ mice, which were kindly provided by Dr. Ho-Young Lee (University of Texas MD Anderson Cancer Center), carry a latent *K-ras* allele with two copies of exon 1, one wild type and the other mutant (G12D). K-ras^+/−^ F1 mice were generated by crossing K-ras^+/−^ heterozygotes with FVB mice. COX-2^−/−^ mice were generated by backcrossing male FVB/COX-2^+/−^ with female FVB/COX-2^+/−^ mice. Female COX-2^+/−^ mice were crossed with male K-ras mice to obtain K-ras/COX-2^+/−^, K-ras/COX-2^+/+^ (K-ras/COX-2 wild type, K-ras), and COX-2^+/−^ mice. Finally, female K-ras/COX-2^+/−^ mice were crossed with male COX-2^−/−^ mice to obtain K-ras/COX-2^−/−^ mice.

Genotypes were determined by polymerase chain reaction (PCR) analysis of genomic DNA derived from the tips of the mice's tails as described previously [29]. In short, mouse tail tips were lysed in Direct PCR Lysis Buffer (Viagon Biotec Inc, Los Angeles, CA) and incubated in 56°C overnight. The mixture was then subjected to PCR with Go Taq Green Master buffer (Promega Inc, Madison, WI). Two primer sets (Sigma Aldrich, St. Louis, MO) were used in separate reactions. Primers 1 and 3 amplified a fraction of the 400-bp product from the mutant allele of K-ras, whereas primers 1 and 2 amplified a 200-bp product from the wild type allele. The primers were: 1) 5′ TGCACAGCTTAGTGAGACCC 3′; 2) 5′ GACTGCTCTCTTTCACCTCC 3′; and 3) 5′ GGAGCAAAGCTGCTATTGGC 3′.

PCR was performed for 5 minutes at 95°C, followed by 32 cycles (30 seconds at 94°C, 30 seconds at 58°C, and 30 seconds at 72°C), followed by a final extension step for 5 minutes at 72°C. The primers for COX-2 were: IMR 013 5′ CTT GGGTGGAGAGGCTATTC 3′; IMR 546 5′ ATCTCAGCA CTGCATCCTGC 3′; and IMR 547 5′ CACCATAGAATCCAGTCCGG 3′. Primer 013 and primer 547 amplified a1.4kb fragment from the mutant allele of COX-2, whereas primers 546 and 547 amplified a 900 bp product from the wild type allele.

### Animals

All transgenic mice (*n* = 10–12 per group) had free access to diet and water and were housed in specific pathogen-free conditions. All animal experiments were approved by the Institutional Animal Care and Use Committee at MD Anderson. Virgin female and male mice were euthanized via CO_2_ at 2, 3.5, or 4 months of age. Mice's lung tissues were perfused and then fixed in 10% buffered formalin (Sigma) for *ex vivo* MRI and histopathological examination. Part of each fixed lung tissue specimen was embedded in paraffin, sectioned, and evaluated by a pathologist to determine the stage of the cancer. Lung tumor tissue was also collected, snap-frozen in liquid nitrogen, and stored at −80°C until further analysis.

### Cell lines

Human non-small cell lung cancer cell A549 cells were obtained from the American Type Culture Collection (Manassas, VA) and maintained in a humidified atmosphere containing 5% CO_2_ at 37°C. A549 cells were routinely growing in DMEM-F12 medium (Invitrogen) supplemented with 10% heat inactivated fetal bovine serum (Hyclone Laboratories Inc., Logan, UT), 50 IU/ml penicillin and 50 μg/ml streptomycin, and 2 mM L-glutamine from GIBCO (Invitrogen). All cell lines were authenticated via microscopic morphology check and DNA characterization. The stably COX-2 knocking down cells were developed by transfecting the A549 cells with COX2 shRNA Lentiviral Particles (Santa Cruz) using the method published previously [16].

### *Ex vivo* MRI

Lung tumor development was further assessed by *ex vivo* MRI. All MRI studies were performed on a 4.7T scanner (Bruker BioSpec, 47/40 USR, Bruker Biospin, Billerica, MA) using a 60-mm gradient insert and a volume resonator with a 35-mm inner diameter. *Ex vivo* lung specimens were suspended in phosphate-buffered saline and placed in 50-ml conical tubes, which were placed on a positioning sled. Orthogonal 3-plane scout scans were acquired for specimen positioning. MR images were acquired using a heavily T1-weighted 3D fast low-angle short spoiled gradient echo sequence (repetition time, 20 ms; echo time, 3.8 ms; flip angle, 25°; field of view = 40 × 30 × 30 mm^3^; image matrix, 256 × 192 × 64; number of signal averages, 4) and a heavily T2-weighted 3D rapid acquisition relaxation enhanced spoiled gradient echo sequence (repetition time, 2000 ms; echo time, 104.5 ms; flip angle, 180°; field of view, 40 × 30 × 30 mm^3^; image matrix, 256 × 192 × 64; number of signal averages, 4). The total scan time for each sequence was about 17 minutes. Lung tumors were counted, and lung tumor volumes were calculated [30].

### Hematoxylin and eosin staining

Hematoxylin and eosin staining was performed in the formalin fixed lung tumors/tissue. Sections of paraffin-embedded specimens were cut at a 5-μm thickness, placed on glass slides, and deparaffinized. The sections were then rinsed and counterstained in eosin-phloxine solution according to a previously described protocol [31].

### Immunohistochemistry

Immunohistochemistry was performed using the lungs from the mice. Lung tissues were fixed with formalin, embedded in paraffin, mounted on glass slides. The slides were incubated with xylene and ethanol overnight at 42°C and then blocked with 3% hydrogen peroxide. After rinsed with water, the slides were placed in citrate buffer (pH 6.0, Diagnostic Biosystems, Pleasanton, CA). Antigen retrieval was then performed. After PBS washing, the slides were washed with phosphate-buffered saline, incubated with a primary antibody against Ki-67 (NeoMarkers, Fermont, CA), and then incubated with a biotinylated secondary antibody (1:200). The slides were treated with avidin-biotinylated horseradish peroxidase complex reagent for 30 min, mounted, and imaged under a microscope. The positive signal was counted and scored [32].

### Liquid chromatography-tandem mass spectrometry

For the assessment of prostaglandin metabolism in mouse lung tissues, frozen lung tumor tissues from 14- to 16-week-old K-ras/COX-2^−/−^ and K-ras mice were homogenized and then analyzed by a liquid chromatography-tandem mass spectrometry (LC-MS/MS) method published previously [33]. A similar procedure was applied to the intracellular prostaglandin analysis in A549 cells without homogenization. The tissue or cellular prostaglandin levels were normalized to the protein concentration of the homogenate.

Tetranor-PGEM, a urinary metabolite of PGE_2_, was extracted and analyzed by LC-MS/MS according to a modified version of the method previously published by Song *et al* [34]. Briefly, 100 μl of mouse urine was derivatized with methoxyamine hydrochloride and incubated for 15 min at room temperature and then applied to a solid-phase extraction cartridge (Waters Oasis HLB 1cc). Samples were reconstituted and then injected into an Agilent 6460 triple quadrupole liquid chromatograph/mass spectrometer for quantitative analysis. PGEM was separated using a C18 analytical column (2 × 100 mm, 3 μm, Kinetex); the mobile phase consisted of 0.05% acetic acid to achieve the best separation. Concentrations of metabolites were quantified using an authentic standard curve and normalized to the amount of creatinine in the urine samples.

### Western blot analysis

Western blotting was performed to determine protein expression in the tissues. Mouse lung tissues were homogenized in ice-cold lysis buffer (Invitrogen, Camarillo, CA) and then immediately subjected to sonication. Protein (50 μg) was separated on a 10–15% sodium dodecyl sulfate gel and analyzed by Western blotting using antibodies against 15-PGDH (Novus Biological Inc., Littleton, CO) and MEK, Erk1/2, and p-Erk1/2 (Cell Signaling Technology, Danvers, MA). β-Actin (Sigma Aldrich Inc.) was used as the loading control. Membranes were blocked in 5% nonfat milk in Tris-buffered saline/0.1% Tween 20 and then probed with primary antibody at 4°C overnight. After washing and incubation with secondary antibodies, the immunoblotted proteins were detected using SuperSignal chemiluminescent substrate (Amersham Biosciences, Piscataway, NJ) according to the manufacturer's instructions.

### Soft-agar colony formation assay

Colony formation assays were performed as described [35, 36]. In brief, A549 or COX-2 knockdown A549 cells (5 × 103) were suspended in 0.35% agarose solution in DMEM/F12 media with 10% FBS, Puromycin 1ug/ml and 1% penicillin/streptomycin over a 0.7% agar layer (60 mm dish). Cells were grown for 21 days at 37°C and 5% CO2. The effect of COX-2 knockdown on the colony formation was evaluated by quantifying the number of colonies formed. The colonies were stained by crystal violet and then counted according to defined size of colony. The colonies larger than 100 mm in diameter were counted.

### Statistical analysis

Data are presented as means ± SDs or SEMs. Differences between K-ras/COX-2^−/−^ mice and K-ras mice were assessed for significance using either the Student *t*-test or Wilcoxon rank-sum test. *p* values less than 0.05 were considered statistically significant.

## SUPPLEMENTARY FIGURES


