# Supplementary material for: Deletion of cyclooxygenase-2 inhibits K-ras–induced lung carcinogenesis
Source: Oncotarget. 2015 Oct 3;6(36):38816–26. doi: 10.18632/oncotarget.5558 (PMC4770739; doi:10.18632/oncotarget.5558)
Supplement: Supplementary file 1 [file oncotarget-06-38816-s001.pdf]

SUPPLEMENTARY FIGURES

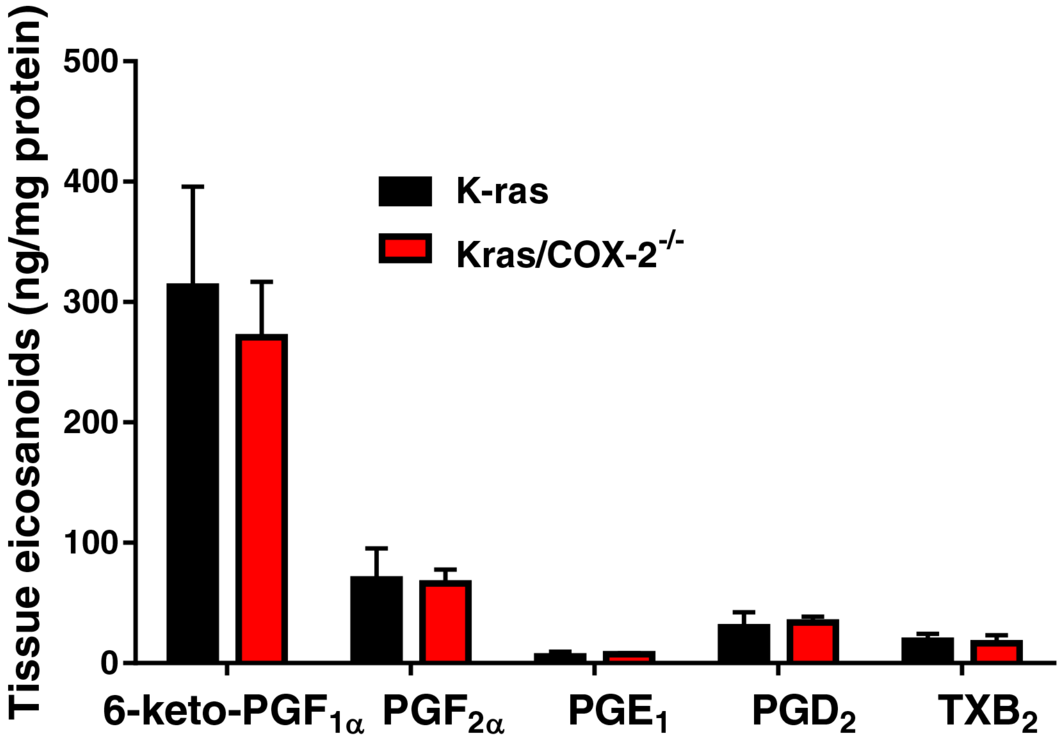

Supplementary Figure S1: Other eicosanoids in tissue from K-ras/COX-2$^{-/-}$ mice and K-ras$^{-/-}$ mice.

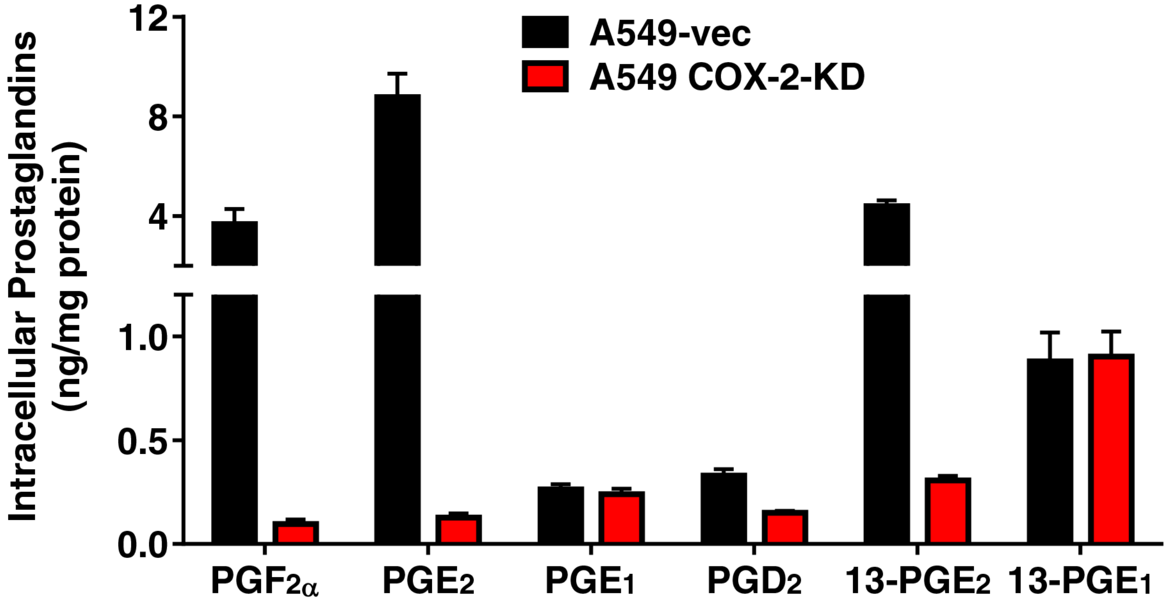

Supplementary Figure S2: Other eicosanoids in A549 cells with and without COX-2 knockdown (KD).
